# Supplementary figures and images for: Biochemical Characterisation of the Short Isoform of Histone N-Terminal Acetyltransferase NAA40
Source: Biomolecules. 2024 Sep 2;14(9):1100. doi: 10.3390/biom14091100 (PMC11430322; doi:10.3390/biom14091100)

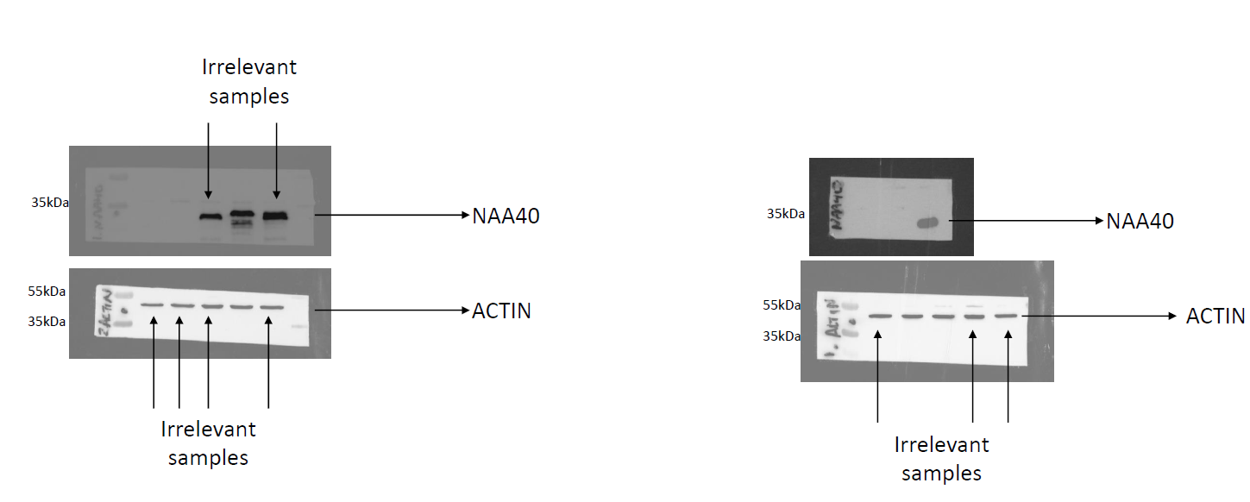

Supplement: Supplementary file 1 [file biomolecules-14-01100-s001.zip › biomolecules-3145428-supplementary-re/FigS1.png]

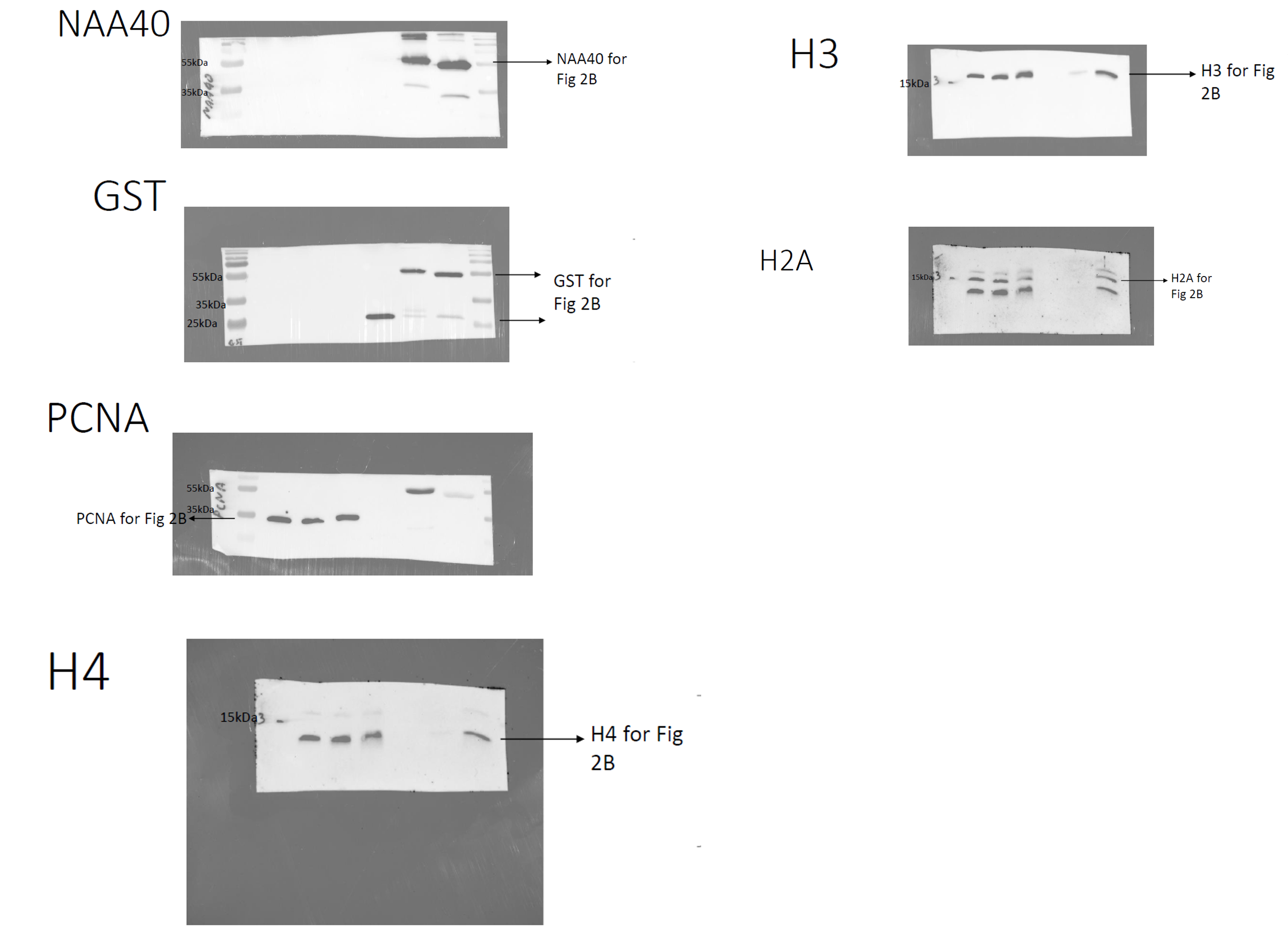

Supplement: Supplementary file 1 [file biomolecules-14-01100-s001.zip › biomolecules-3145428-supplementary-re/FigS2.png]

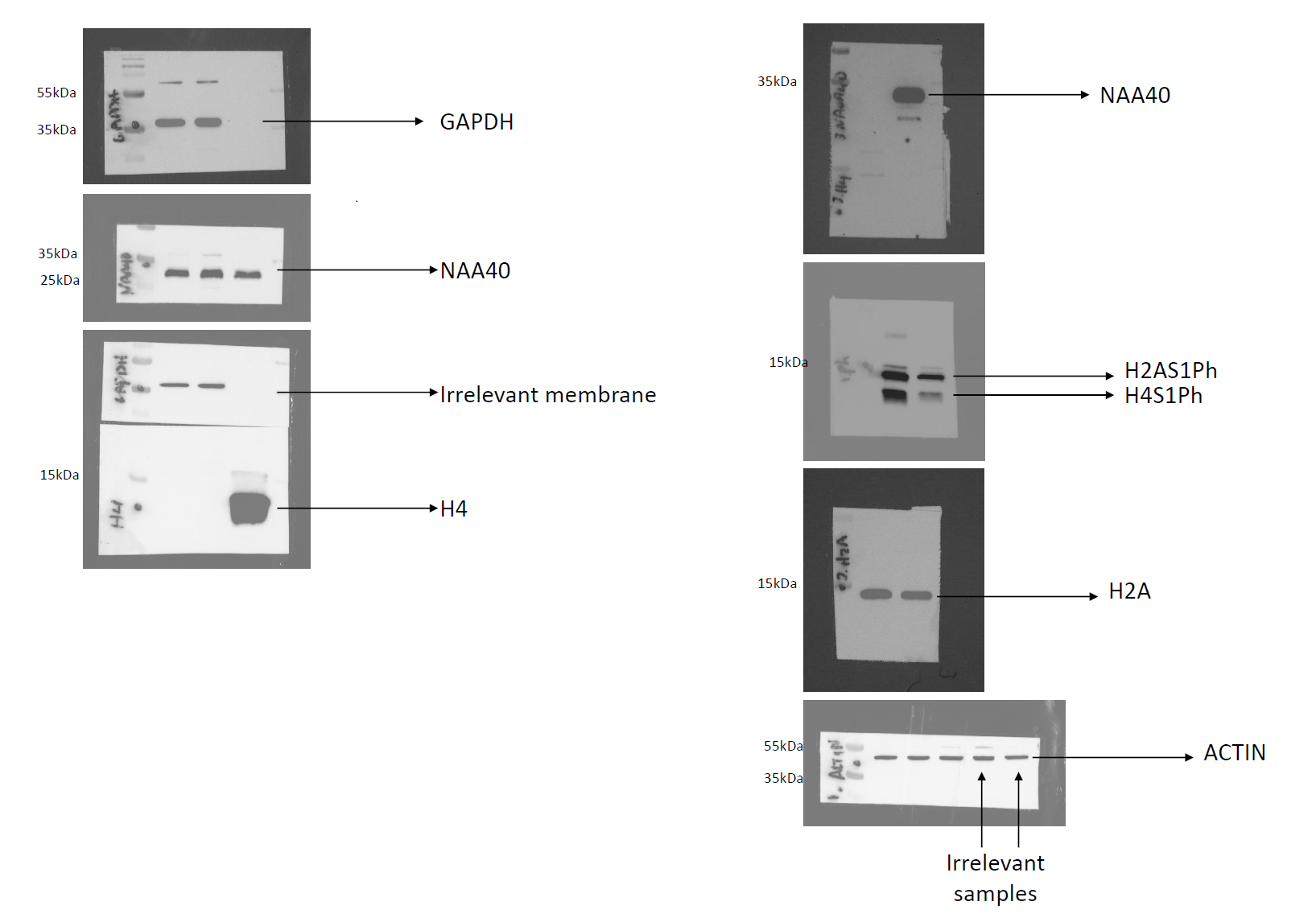

Supplement: Supplementary file 1 [file biomolecules-14-01100-s001.zip › biomolecules-3145428-supplementary-re/FigS3.png]
